# Clinical implication of coronary artery calcium score

**DOI:** 10.1002/clc.23614

**Published:** 2021-05-06

**Authors:** Teruhiko Imamura

**Affiliations:** ^1^ Second Department of Internal Medicine University of Toyama Toyama Japan

To the Editor

Huang and colleagues demonstrated that coronary artery calcium score (CAC) improved coronary artery disease (CAD) risk prediction, compared to conventional risk scoring, even in the absence of cardiovascular risk factor screening.^1^ Several concerns should improve their findings.

The authors discussed the cost‐effectiveness. The cost of CAC is cheaper than that of computed tomography coronary angiogram. Given that approximately 65% of their study population had risk scores <10% after application of CAC, many patients can avoid unnecessary computed tomography coronary angiogram and save costs without detailed cardiovascular risk factor screening.^1^ However, in real‐world practice, detailed interviews of chest pain and cardiovascular risk factor screening are useful to plausibly exclude the existence of CAD without further examinations including CAC.^2^ How do the authors consider the importance of detailed interview?

The authors stated that the CAC‐incorporated risk models had approximately comparable sensitivity and specificity to functional stress tests in predicting CAD.^1^ However, the definition of CAD might be different between the two tests. The former defined CAD as coronary artery stenosis >50% observed in computed tomography coronary angiogram, whereas the latter probably defined CAD as functionally‐demonstrated coronary stenosis, which would be more severe.

Lastly, some patients might have any previously implanted devices including pacemaker and cardiac resynchronization therapy with/without a defibrillator. Accurate calculation of CAC might be challenging due to artifact and the prognostic impact of CAC might decrease in such a cohort.

## References

[1] Huang W, Lim LMH, Aurangzeb AS, et al. Performance of the coronary calcium score in an outpatient chest pain clinic and strategies for risk stratification. Clin Cardiol. 2021;44:267‐275. 10.1002/clc.23539. 33434373 PMC7852173

[2] Levine GN, Bates ER, Bittl JA, et al. 2016 ACC/AHA guideline focused update on duration of dual antiplatelet therapy in patients with coronary artery disease: a report of the American College of Cardiology/American Heart Association task force on clinical practice guidelines: an update of the 2011 ACCF/AHA/SCAI guideline for percutaneous coronary intervention, 2011 ACCF/AHA guideline for coronary artery bypass graft surgery, 2012 ACC/AHA/ACP/AATS/PCNA/SCAI/STS guideline for the diagnosis and management of patients with stable ischemic heart disease, 2013 ACCF/AHA guideline for the management of ST‐elevation myocardial infarction, 2014 AHA/ACC guideline for the management of patients with non‐ST‐elevation acute coronary syndromes, and 2014 ACC/AHA guideline on perioperative cardiovascular evaluation and management of patients undergoing noncardiac surgery. Circulation. 2016;134(10):e123‐e155. 27026020 10.1161/CIR.0000000000000404

